# Medaka insulin-like growth factor-2 supports self-renewal of the embryonic stem cell line and blastomeres *in vitro*

**DOI:** 10.1038/s41598-017-00094-y

**Published:** 2017-03-06

**Authors:** Yongming Yuan, Yunhan Hong

**Affiliations:** Department of Biological Sciences, National University of Singapore, Singapore, 117543 Singapore

## Abstract

Insulin-like growth factors (IGFs) regulate diverse processes including energy metabolism, cell proliferation and embryonic development. They activate the IGF signaling pathway via binding to cell surface receptors. Here we report an essential role of IGF2 in maintaining the pluripotency of embryonic stem (ES) cell from medaka (*Oryzias latipes*). The medaka *igf2* gene was cloned for prokaryotically expression of IGF2 ligand and green fluorescent protein-tagged IGF2 namely IGF2:GFP. With flow cytometry analysis, we demonstrated that the IGF2:GFP can bind to the cultured ES cells from medaka and zebrafish respectively. We also verified that IGF2 is able to activate the phosphorylation of Erk1/2 and Akt, and sustain the viability and pluripotency of medaka ES cells in culture. Furthermore, we characterized the binding of IGF2:GFP to freshly isolated blastomeres by fluorescence microscopy and electron microscopy. Most importantly, we revealed the important role of IGF2 in supporting the derivation of blastomeres in short-term culture. Therefore, Medaka IGF2 is essential for the self-renewal of cultured ES cells and blastomeres from fish embryos. This finding underscores a conserved role of the IGF signaling pathway in stem cells from fish to mammals.

## Introduction

The insulin-like growth factors play an important role in the regulation of fetal and postnatal growth in all vertebrates^1, 2^. This complex system includes the ligands of insulin-like growth factors I and II (IGF1 and IGF2) along with the IGF-binding proteins (IGFBPs) and cell-surface receptors consisting of type I (IGF-1R) receptor, type II (IGF-2R) receptor and insulin receptor (IR)^3^. IGF1 and IGF2 are single-chain polypeptide growth factors remarkable conserved through evolution. They exert effects on the target cells via binding on the receptors of IGF-IR, IR or IGF-2R to trigger their intrinsic tyrosine kinase domain activities^4^ and subsequently activate the PI3K/Akt pathway^5, 6^ and MAPK/Erk pathway^7, 8^.

IGF2 is a short peptide of 67 to 70 amino acids consisting of 4 domains (B, C, A and D). It was synthesized as preprohormone containing an E domain at the C-terminus and a signal peptide at the N-terminus. These two domains are post-translationally cleaved to generate the mature peptide of IGF2 ligand with bioactivity^9^. IGF2 is mainly produced in the liver and it regulates the cell metabolism, growth and pluripotency^10, 11^. In fish, since the first identification of IGF2 mRNA in Rainbow trout (*Oncorhynchus mykiss*)^12^, the IGF2 mRNA has been detected in different organs and at all development stages in a wide variety of species such as rabbitfish (*Siganus guttatus*)^13^, tilapia (*Oreochromis mossambicus*)^14^, zebrafish (*Danio rerio*)^15^ and carp (*Cyprinus carpio*)^16^. The recombinant IGF2 derived from fish species such as salmon (*Salmo salar*)^17^, turbot (*Psetta maxima*)^18^ and rainbow trout (*Oncorhynchus mykiss*)^19^ has been prokaryotically expressed. Besides the physiological function analysis, the cell surface binding ability of IGF2 was also assayed with the directly labeling such as radioactive ^125^I or with the modified antibody to enlarge the binding signal^18, 20^.

Medaka is a laboratory fish and holds many genetic resources and toolboxes to study their functions in cellular processes. More specifically, medaka has given rise to haploid embryonic stem (ES) cell line such as HX1 that is capable of whole animal production by semicloning^21^. In this study, we first time cloned and expressed the recombinant IGF2 of medaka. With the help of enhanced green fluorescent protein (EGFP) fused to IGF2, the binding of IGF2:GFP to cultured embryonic stem cells of medaka and zebrafish was confirmed by flow cytometry analysis. The cell viability assay and gene transcription analysis further verified that the IGF2 can promote the cellular health and sustain the pluripotency of medaka ES cells. Moreover, we detected IGF2 binding to the blastomeres under fluorescence microscopy and transmission electron microscopy (TEM). Most importantly, we revealed that the IGF2 can sustain the viability of blastomeres and support the derivation of blastomeres in short-term culture.

## Results

### Gene cloning

**Figure 1:**
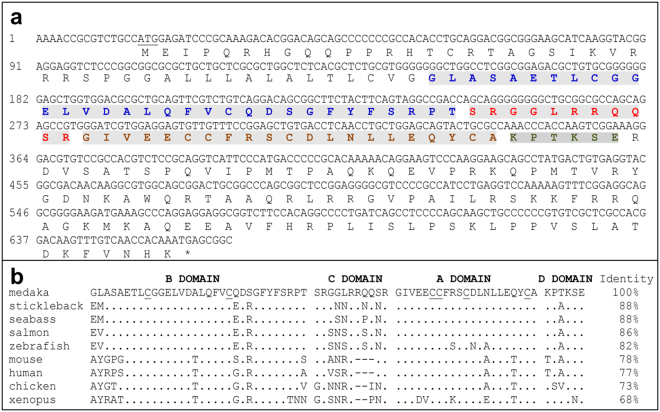
Nucleotides and predicted amino acid sequence alignment of medaka IGF2. (**a**) Nucleotides and predicted domains of medaka IGF2. Nucleotides are numbered to the left. Translated amino acids are shown below the DNA sequence. Mature peptide consisting B, C, A and D domains are distinguished with blue, red, brown and cyan in shadowed letters respectively. Signal peptide locates before mature peptide and E domain is after D domain. (**b**) Sequence alignment of IGF2 mature peptides. Identical positions are indicated as dots, and gaps (dashes) are introduced to optimize alignment. Six conserved cysteine residues are underlined. Accession numbers of the IGF2 sequences are ENSGACP00000014712 for stickleback (*Gasterosteus aculeatus)*, AAB64195.1 for seabass (*Lates calcarifer)*, CAA65862.1 for salmon (*Oncorhynchus keta*), NP_001001815.1 for zebrafish (*Danio rerio*), NP_034644.1 for mouse (*Mus musculus*), NP_001121070.1 for human (*Homo sapiens*), NP_001025513.1 for chicken (*Gallus gallus*) and NP_001082128.1 for xenopus (*Xenopus laevis*).

With primer pair of IGF2F plus IGF2R, a 636 bp DNA fragment was PCR-amplified from the medaka fish cDNA template. Its translation generates the prepro-IGF2 consisting of an N-terminal signal peptide, mature peptide namely IGF2 and an E-domain at C-terminus (Fig. 1a). After the post-translational proteolysis, the signal peptide and E-domain were removed to generate a 70-amino acids protein of IGF2 consisting of 4 domains (B, C, A and D). In the IGF2 mature peptide, the six characteristic cysteine residues (Cys^B9^, Cys^B21^, Cys^A6^, Cys^A7^, Cys^A11^, and Cys^A20^) involved in the formation of disulfide bounds in IGF2 are totally conserved among medaka and the listed species (Fig. 1b). The medaka IGF2 exhibits an identity of 82% to zebrafish and 77% to human respectively (Fig. 1b).

### Protein expression and purification

**Figure 2:**
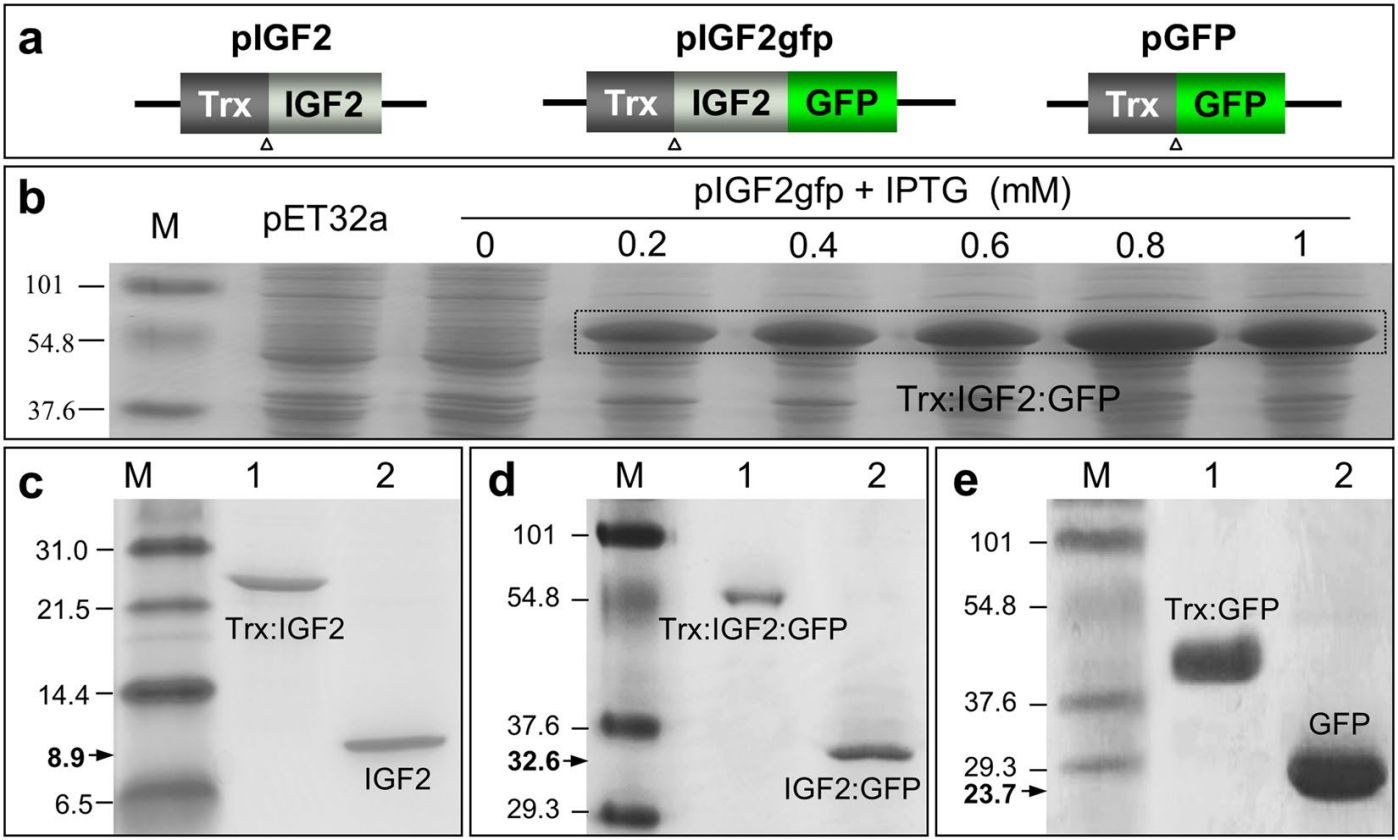
Production of recombinant proteins. (**a**) Schematic map of vectors expressing IGF2 (pIGF2), IGF2:GFP (pIGF2gfp), and GFP (pGFP). Trx, thioredoxin protein tag; Triangle shows the enterokinase cleavage site. (**b**) Expression of IGF2:GFP with IPTG induction. BL21 strains transfected with pIGF2gfp was treated with IPTG at concentration ranged from 0.2 to 1 mM. Expression of Trx-tagged IGF2:GFP was examined with SDS-PAGE. Target proteins were highlighted with box. (**c–e**) Purification and cleavage of recombinant proteins. SDS-PAGE showing the Trx tag was removed from purified recombinant protein IGF2 (**c**), IGF2-GFP (**d**), and GFP (**e**).

We constructed plasmids pIGF2 and pIGF2gfp to express IGF2 and IGF2:GFP respectively, and another plasmid pGFP encodes GFP protein as a control (Fig. 2a). After transforming BL21 strains with constructs, we optimized the IPTG induction concentration ranged from 0.2 to 1 mM. When the culture was induced with 0.8 mM IPTG overnight at 18 °C, the yield of IGF2:GFP was maximized to about 15 mg per liter of cell culture after Ni-NTA agarose purification (Fig. 2b). The IGF2 and GFP were also expressed successfully at the similar condition (Fig. 2c and  e). Then the N-terminal fused tag in recombinant protein was digested by enterokinase and captured with S-tag affinity agarose to give the final products of IGF2 (8.9 KDa, Fig. 2c), IGF2:GFP (32.6 KDa, Fig. 2d) and GFP (23.7 KDa, Fig. 2e) respectively.

### IGF2:GFP binds to stem cells in culture

**Figure 3:**
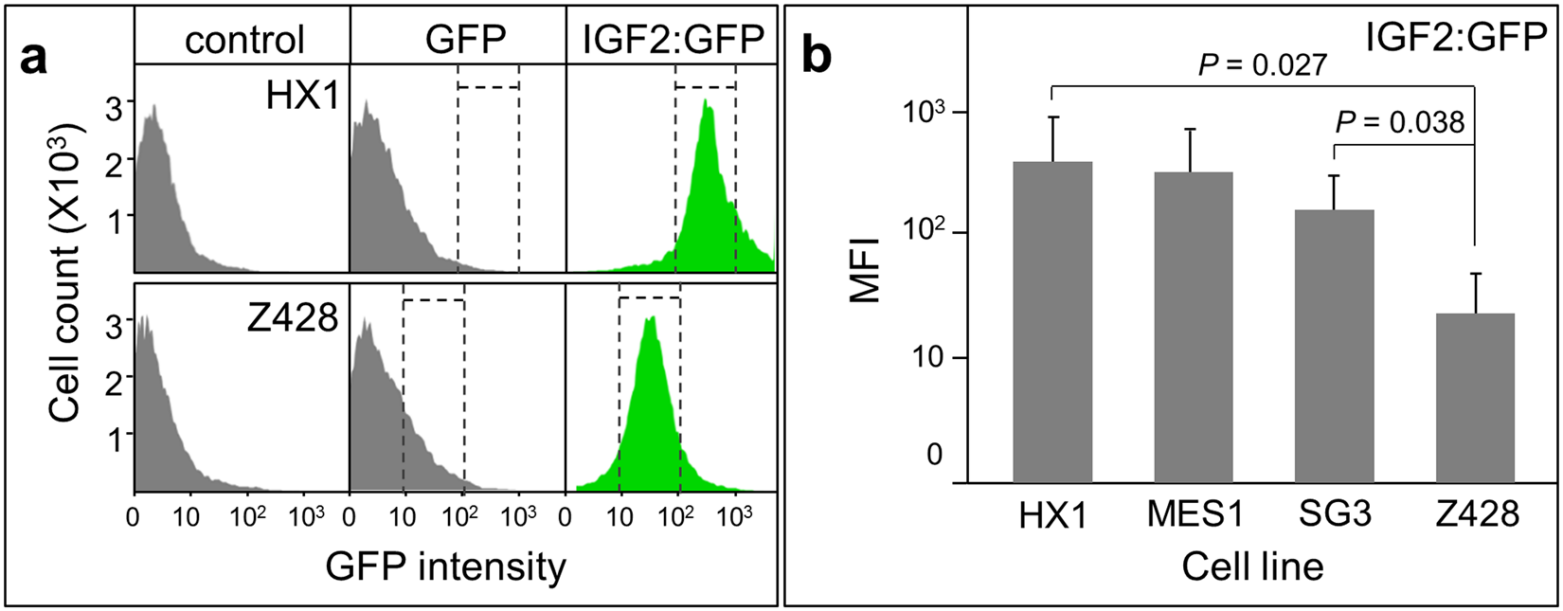
IGF2:GFP binding to stem cells. IGF2:GFP was incubated with medaka or zebrafish cell lines and the fluorescence of ligand was analyzed with flow cytometry. (**a**) Flow cytometric analysis of IGF2:GFP binding to medaka ES cell HX1 and zebrafish ES cell Z428. (**b**) Binding ability of IGF2:GFP to the stem cells presented by mean fluorescence intensity (MFI).

Totally 4 stem cell lines were used in this study, including medaka cell lines HX1, MES1 and SG3 and zebrafish cell line Z248. Cultured cells were starved in the basic medium before the adding of IGF2:GFP. After ligand incubation and following wash, the cells were stained with Hoechst 33342 and subsequently examined with a flow cytometer. When the cells were incubated with IGF2:GFP, flow cytometry histograms showed the specific binding of IGF2:GFP comparing to the cells incubated with or without GFP, which was represented in cell culture of HX1 and Z248 (Fig. 3a). Meanwhile, the amount of IGF2:GFP bond to the medaka cell lines was significantly higher than the amount to zebrafish cell, which was evidenced by the significant difference of fluorescence intensity between each group (Fig. 3b). Taken together, the IGF2:GFP not only specifically binds to the medaka stem cell but also to the zebrafish stem cell, demonstrating a cross-species binding activity of this ligand.

### IGF2 supports self-renewal of medaka ES cells

**Figure 4:**
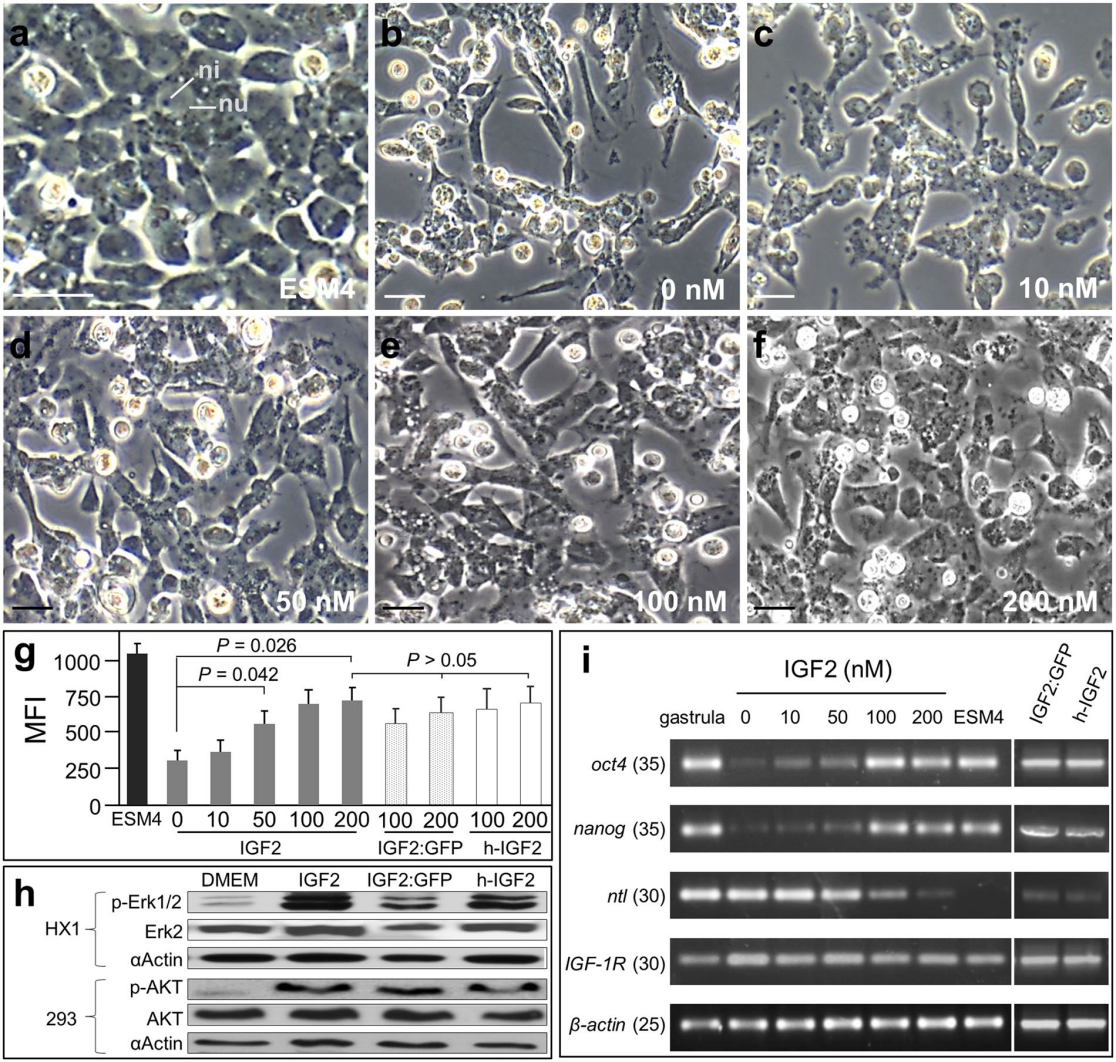
IGF2 supports ES cells self-renewal. HX1 cells were cultured for 2 days in basic medium with growth factors added at varying concentrations. The morphology, viability and transcription profiles of cultured cells were examined. (**a**–**f**) Micrographs of HX1 cells cultured in ESM4 or in basic medium containing IGF2 at indicated concentration. nu, nucleus; ni, nucleolus. Scale bars, 5 μm. (**g**) Viability of cells cultured in ESM4 or medium with supplemented growth factors. Cell viability was measured with reagent of AlamarBlue and represented by MFI. (**h**) Western blot analysis, showing the phosphorylation of Akt activated by IGF2 in 293 cells. Starved HX1 and 293 cell were incubated with IGF2, IGF2:GFP and h-IGF2 respectively at the concentration of 200 nM respectively. The phosphorylation of Erk1/2 was examined with phospho-Erk1/2 antibody (p-Erk1/2), and the phosphorylation of Akt was detected by phospho-Akt antibody (p-Akt). Antibody against Erk2, Akt and Actin was used to identify the intracellular target respectively. (**i**) RT-PCR analysis of gene transcription. Gastrula embryos were used as positive control, and β-actin is internal control. Numbers of PCR cycles are given in parenthesis.

After confirming that the IGF2 can bind to ES cells in culture, we furthered our study to illustrate the bioactivity of this ligand. The medaka stem cell lines were routinely maintained in ESM4 which contains various growth factors from the supplemented FBS, fish serum and embryo extracts. To test the bioactivity of IGF2, HX1 cell were first starved in the basic medium for 8 h to eliminate the effects of existing growth factors, and they were subsequently cultured for 2 days in the medium containing IGF2 added at different concentrations. We examined the morphology, viability, signaling pathway activation and pluripotency of HX1 cells under these defined culture conditions. In ESM4 medium, HX1 cells displayed a round shape, little cytoplasm and prominent nuclei with large nucleoli (Fig. 4a), which has been previously reported^21^. But when HX1 cells were cultured in the DMEM basis medium as a control, cell morphology changed remarkably as evidenced by the clustering of elongated cells (Fig. 4b). The elongated cells were also found in medium containing IGF2 ranged from 10 nM to 200 nM (Fig. 4c–f). But comparing to the control group, the morphology of the cultured cell was closer to the stem cell in ESM4, especially when they were cultured with supplemented IGF2 of 200 nM (Fig. 4f). Meanwhile, the change of cell morphology was also found in the basic medium containing IGF2:GFP or human IGF2 at the concentration of 100 nM and 200 nM respectively (Fig. S1).

To further evaluate the bioactivities of IGF2 quantitatively, AlamarBlue was added into the culture medium, and the fluorescence intensity was used to assess the viability of the cells as reported previously^22^. When cultured in defined medium containing IGF2 at different concentrations, the viability of HX1 cells was apparently lower than the cells cultured in ESM4 (Fig. 4g). However, the supplemented IGF2 demonstrated its activity to promote the cellular viability, which is evidenced when IGF2 was added at 50 nM or even higher concentration until 200 nM (Fig. 4g). Similarly, after IGF2:GFP or h-IGF2 was added at the concentration of 100 or 200 nM respectively, the cellular viability increased remarkably than the control (Fig. 4g). But there was no significant difference of the cellular viability in medium containing the above growth factors at 200 nM (Fig. 4g), indicating IGF2:GFP and h-IGF2 also exhibited the similar activity as IGF2 to promote the cellular health of HX1.

Additionally, we examined the signaling pathways activated by medaka IGF2 by detecting the phosphorylated intracellular proteins with western blot analysis. Commercial antibody was introduced to detect the phosphorylated Erk (p-Erk1/2) involved in MAPK/Erk signaling pathway and phosphorylated Akt (p-Akt) in PI3K/Akt pathway respectively. To test the specificity of these antibodies to medaka fish cell protein. HX1 and 293 cells cultured in complete medium and basic DMEM medium were sampled for western blot analysis respectively. It showed that the Erk2, p-Erk1/2 and Akt in HX1 and 293 cells can be detected specifically by the related antibody respectively. But the antibody against p-Akt can only detect the phosphorylated Akt in 293 cells (Fig. S2). Thus, we examined the phosphorylated Erk1/2 after the HX1 cells were incubated with growth factor of IGF2, IGF2:GFP and h-IGF2 respectively. It showed that there was significant increase of the p-Erk1/2 after growth factors incubation, indicating the medaka IGF2 and human IGF2 can activate the MAPK/Erk pathway (Fig. 4h) in HX1 cell. Antibody against p-Akt was used to detect the phosphorylation of Akt in 293 cells after growth factor treatment. It showed that the medaka IGF2 as well as the GFP tagged IGF2 have the similar function as human IGF2 in activating the PI3K/Akt pathway in 293 cells, as the increase of p-Akt was detected clearly in the 293 cells incubated with medaka IGF2, IGF2:GFP and h-IGF2 respectively (Fig. 4h).

Furthermore, we examined the pluripotency of cultured cells with RT-PCR by detecting the transcription of pluripotency genes of *nanog* plus *oct4* and a differentiation marker namely *ntl*. When HX1 cells were cultured in medium without or with IGF2 at concentrations of 10 nM and 50 nM, the transcription of *nanog* and *oct4* obviously decreased comparing to the cells cultured in ESM4. Meanwhile, the transcription of *ntl* was apparently up-regulated (Fig. 4i). However, when IGF2 was added at 100 nM or higher concentration of 200 nM, the transcriptions of *nanog* and *oct4* were up-regulated, and transcription of *ntl* decreased remarkably but still detectable (Fig. 4i). When IGF2:GFP and h-IGF2 was added at the concentration of 200 nM respectively, the transcriptions level of *nanog* and *oct4* were similar to the cells cultured in medium with IGF2. Meanwhile, the transcription of *ntl* also decreased significantly comparing to the cells in basic medium. The transcription of IGF-1R exhibits a stable level in all of the tested cells (Fig. 4i). Taken together, the medaka recombinant IGF2 can support the self-renewal of medaka ES cell but not sufficient.

### IGF2:GFP binds to medaka blastomeres

**Figure 5:**
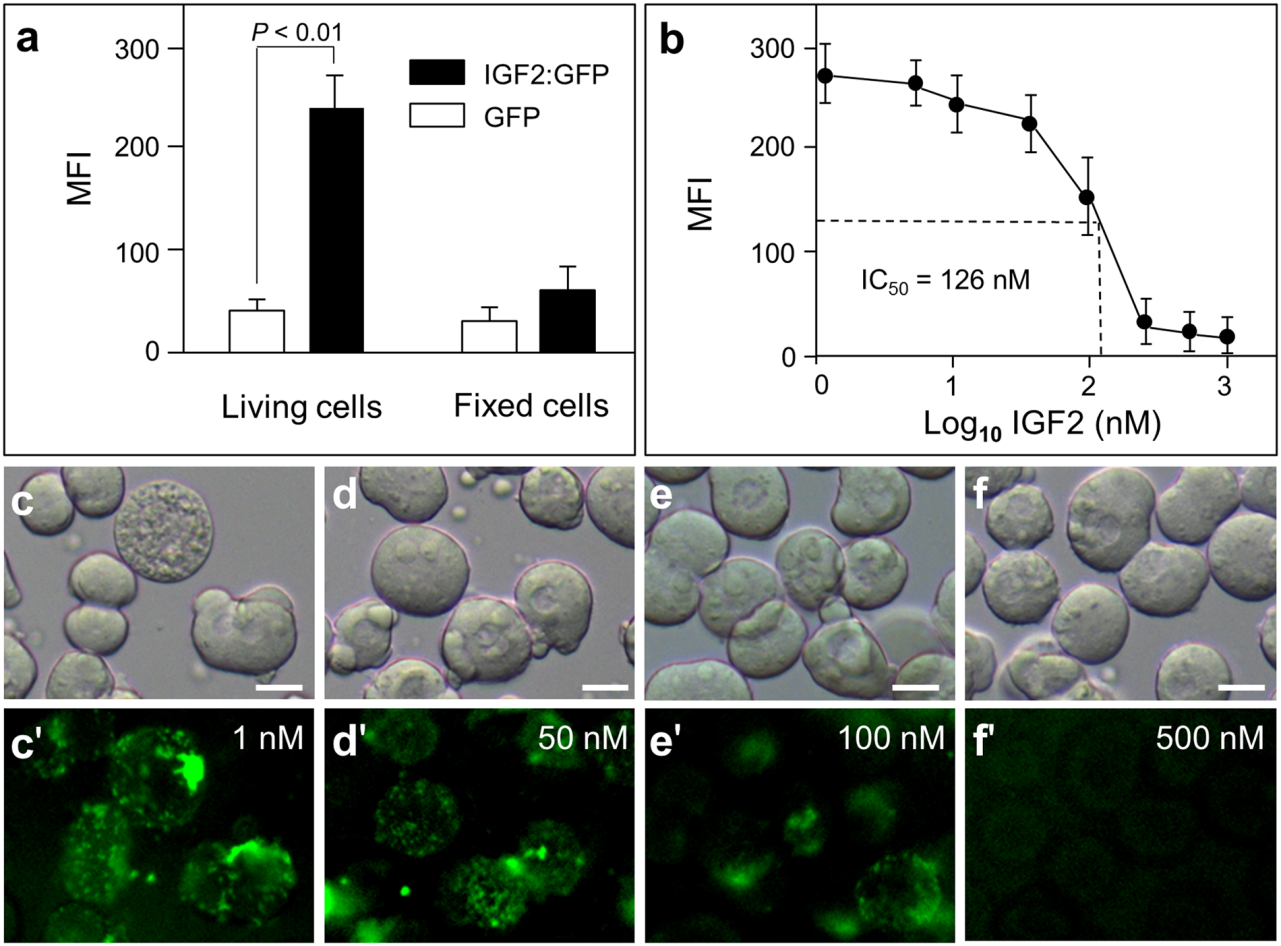
IGF2:GFP binds to madaka blastomeres. (**a**) Relative binding ability of IGF2:GFP. Live or fixed medaka blastomeres were incubated with IGF2:GFP. MFI in micrographs was calculated to evaluate the binding ability of IGF2:GFP comparing to control protein GFP. (**b**) Competitive binding assay. Blastomeres were co-incubated with IGF2:GFP (200 nM) and IGF2 at indicated concentration. Fluorescence intensity was measured to determine the competitive binding curve. (**c–f′**) Phase contrast and fluorescence micrographs of blastomeres representing the competitive binding of IGF2:GFP and IGF2 at indicated concentration. Top, phase contrast; bottom, fluorescence optics. Scale bars, 5 μm.

After verifying the bioactivity of IGF2 to ES cells in culture, we also tested the binding of IGF2:GFP to the cells in medaka embryos. The medaka blastomeres were isolated from embryos and incubated with IGF2:GFP at the concentration of 100 nM. After washing with PBS, the blastomeres were checked under fluorescence microscopy and the mean fluorescence intensity on each cell was calculated to evaluate the binding ability of tested protein. It revealed that the IGF2:GFP can specifically bind to living blastomeres comparing to control protein of GFP, but not to the fixed cells (Fig. 5a). Subsequently, we co-incubated blastomeres with IGF2:GFP and IGF2 for competitive binding assay. The fluorescent intensity curve revealed that when the concentration of IGF2 increased in the incubation buffer, the fluorescent intensity decreased accordingly, indicating that the binding sites on the surface of blastomeres were competitively occupied by IGF2 (Fig. 5b). Furthermore, the half inhibitory concentration (IC_50_) was calculated from the competitive binding curve with a value of about 126 nM (Fig. 5b). From the represented micrographs of GFP signals on blastomeres, we can also detect that the fluorescence intensity is lower when blastomeres were incubated with higher concentrations of IGF2 (Fig. 5c′–f′). Taken together, the IGF2:GFP can specifically bind to ES cells in culture and blastomeres from medaka embryo.

### Locate the IGF2 on the surface of blastomeres

**Figure 6:**
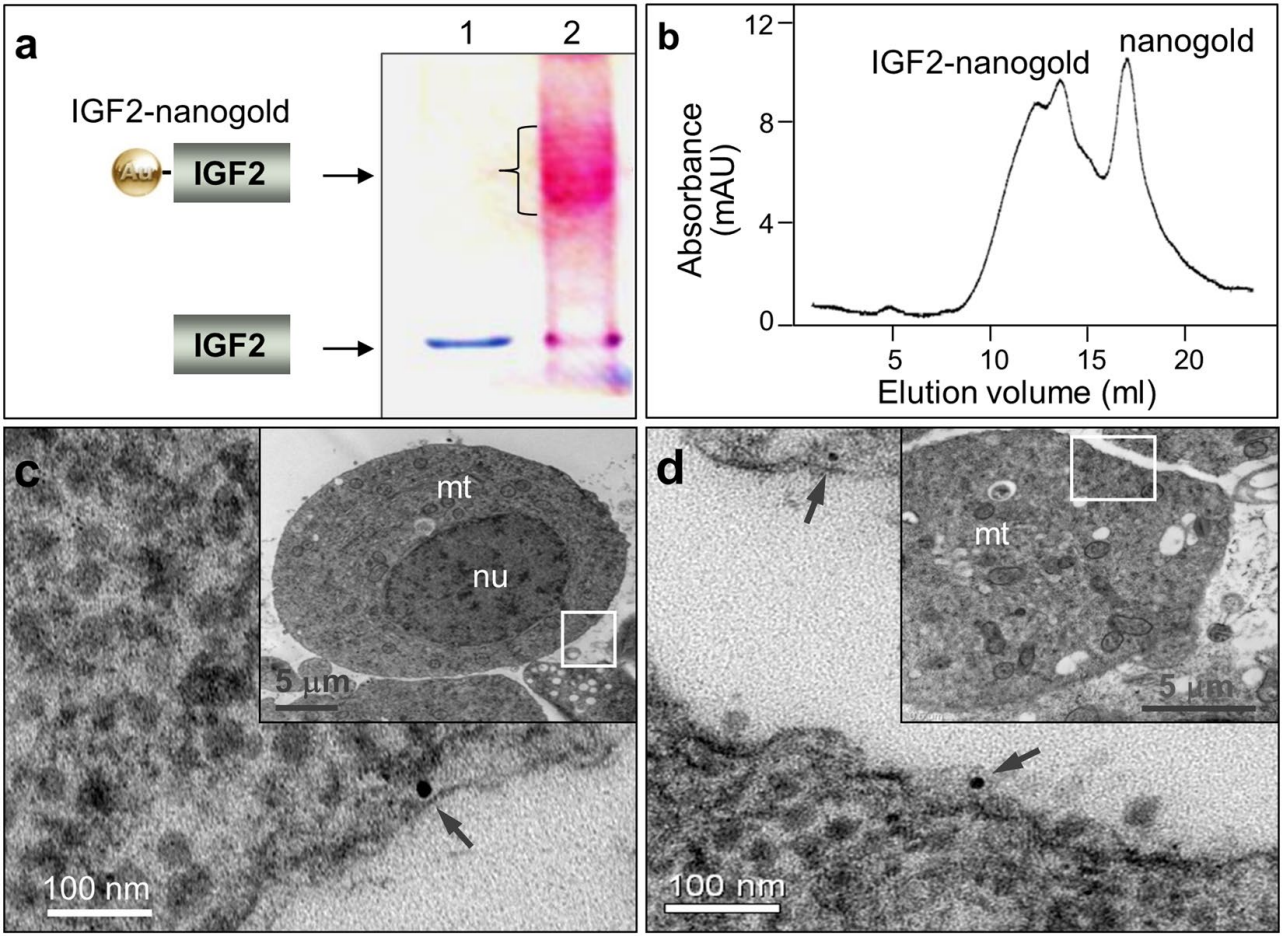
Location of IGF2 on the cell membrane. IGF2 was conjugated with nanogold and incubated with medaka blastomeres. Under electron microscopy, the ligand was detected on the cell surface or beneath the membrane. (**a**) 10% SDS-PAGE analysis of non-reduced and unboiled IGF2 (lane 1) and IGF2-Nanogold conjugate (lane 2). IGF2 was incubated with 1.4 nm nanogold, and the production was analyzed with SDS-PAGE. The protein band migrated at a higher apparent molecular weight after incubation with Nanogold (lane 2), demonstrating covalent attachment of 1.4 nm nanogold to IGF2. (**b**) Purification of IGF2-Nanogold conjugate with gel filtration. (**c** and **d**) Location of IGF2-nanogold under TEM, showing IGF2-nanogold (arrow) binding to cell. Insets depict the nucleus (nu), mitochondrial (mt) and location of enlarged areas of cells (boxes).

As a growth factor, binding to the receptors on the cell surface is the first step to trigger the signaling pathway. We further analyzed the location of IGF2 by TEM after it was incubated with blastomeres. The IGF2 was incubated with conjugated with 1.4 nm gold particles and the product was verified by SDS-PAGE electrophoresis and subsequently staining. The IGF2-nanogold conjugates migrated slower than IGF2 and exhibited a smear band of higher molecular weight on the gel (Fig. 6a). After staining with Pageblue and gold enhancement reagent, the IGF2 was visualized as a blue band (Fig. 6a, lane 1), but the IGF2-nanogold band was stained into red or purple as a result of the gold deposition (Fig. 6a, lane 2). Subsequently, the IGF2-nanogold and access nanogold was separated with FPLC gel filtration (Fig. 6b). The purified IGF2-nanogold was incubated with blastomeres at 4 °C and then sampled for TEM observation. Under TEM, we can clearly detect the gold particles on the surface of cells or beneath the cell membrane (Fig. 6c and d) but we didn’t find the specific binding of nanogold in control group. It precisely presented the binding of IGF2 to the blastomeres at nanoscale resolution.

### IGF2 supports the derivation of blastomeres

**Figure 7:**
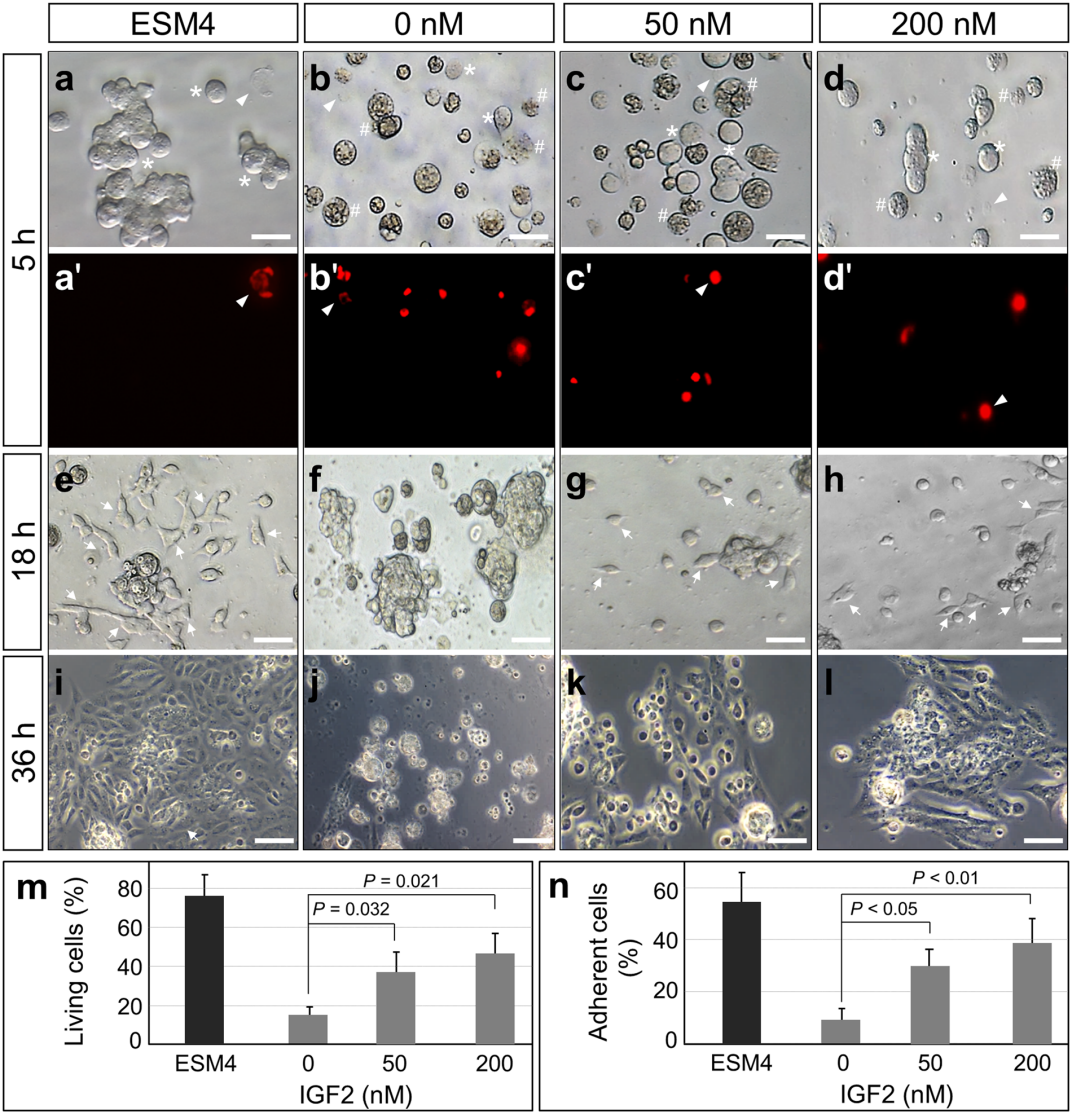
IGF2 supports the derivation of blastomeres in short-term culture. (**a–d′**) Micrographs and PI staining of madaka blastomeres cultured for 5 h. The madaka blastomeres were cultured in ESM4 or defined medium containing IGF2 at indicated concentration. The morphology of cells was recorded with phase contrast microscopy (**a–d**) to identify the living cells (asterisk) and necrotic/dead cells (hash). The dead cells and ghost cells (arrow head) were identified with PI staining (**a′–d′**). (**e–h**) Phase contrast micrographs of blastomeres cultured for 18 h, showing the attached cells (arrow) in primary culture. (**i–l**) Blastomeres cultured for 36 h, showing the derivation of cells. (**m**) Survival rate of blastomeres cultured for 5 h in medium containing IGF2 at indicated concentration. (**n**) Percentage of adherent cells in primary culture at 18 h, showing IGF2 promotes the cell viability and attachment. Scale bars, 20 μm.

From the above results, we confirmed the IGF2 can bind to the surface of blastomeres. We furthered our study to test the bioactivity of IGF2 in embryonic cells primary culture. The blastomeres were isolated from embryos and cultured in medium containing IGF2 ranged from 0 to 200 nM. After incubation for 5 h, the blastomeres were checked with microscopy. Under phase contrast microscopy, the living blastomeres exhibited a round sphere with white color because of low transparency (Fig. 7a–d, asterisk). However, the necrotic/dead cells are more transparent because of the leakage of cytoplasm and blebbing in the cells (Fig. 7b–d, hash). Meanwhile, some ghost cells were not easily observed under light microscopy but detectable with PI staining (Fig. 7a–d′, arrowhead). With the help of phase contrast microscopy and PI staining, the living cells and dead cells were counted for the survival rate calculation. The blastomeres survival rate is about 38% and 57% when incubated in medium containing IGF2 of 50 nM and 200 nM respectively. However, the survived blastomeres in medium without IGF2 were significantly lower (Fig. 7m). After the blastomeres were cultured for 18 h, attached cells were detected in the medium containing IGF2 of 50 nM and 200 nM respectively (Fig. 7g and h, arrow). But the cell attachment seldom occurred when blastomeres were incubated in medium without IGF2, and most of the blastomeres were dead under this culture condition (Fig. 7f). Accordingly, the percentage of attached cells in different culture medium was calculated. It indicated that the attachment ratio of blastomeres cultured in medium containing 50 nM IGF2 is about 33%, and it increased to 39% in medium containing 200 nM IGF2 (Fig. 7n). But in the medium without IGF2, the attached cells were remarkably less (Fig. 7n). After the following culture for 36 h, the derived cell clusters were detected in the medium containing IGF2 (Fig. 7k and l) but not in the medium without IGF2 (Fig. 7j). Taken together, the IGF2 added in medium sustained the viability of blastomeres and supports the cell derivation in short-term culture.

## Discussion

In this study, we cloned the gene encoding medaka IGF2. Its amino acid sequence contains all the features of IGF2 peptides with B, C, A, and D domains and the conservation of the 6 cysteine residues involved in the tertiary structures. The homologies of B and A domains of IGF2 among bony fish are very high, which can be explained that most residues of these domains are involved in the binding of its receptor or IGFBP reported in mammals, such as the Arg^21^ and Phe^23^-Tyr^24^-Phe^25^ motif in domain B and Phe^51^ and Ser^53^ in domain A^23, 24^.

Subsequently, we successfully expressed and purified the recombinant IGF2 and IGF2:GFP under optimized culture condition for further study. The binding of IGF2:GFP to the stem cells of medaka and zebrafish were verified by flow cytometer. It revealed that IGF2:GFP bind not only to the medaka ES cells but also to the ES cells from zebrafish, exhibiting a cross-species binding activity, which could be explained with the high conservation of the IGF2 within different species. It has been reported that the cross-reactivity indeed exists as the recombinant human IGF-I and IGF-II induced the maturation of zebrafish ovarian follicles *in vitro* ^25^. Similar cross-species activity was found in the commercial product of mature human IGF-I (Cat. 05-0111, GIBCO) and IGF-2 (Cat. 14-8505, eBioscience), which have a high identity with the growth factors in bovine, porcine and mouse. Meanwhile, the binding signal of IGF2:GFP on SG3 cells is lower than the signals on HX1 and MES1 cells, which could be explained by the different origins of these cells. HX1 and MES1cells are derived from embryonic stem cells, but SG3 are spermatogonial stem cells. As IGF2 can bind to IGF1R, IGF2R and IR, to overexpress or knockdown center receptors and quantitate the binding signals of IGF2:GFP to these cells will help to identify the binding affinity of this ligand to individual receptor.

Furthermore, we examined the morphology, cellular health and pluripotency of the medaka ES cell HX1 cells cultured in defined medium with supplemented IGF2. It revealed that IGF2 can promote the cell viability and sustain the self-renewal of the ES cells. Similar results were reported in human ES cell culture^11^ and neural stem/progenitor cells^10^. Previous reports indicated that the IGF2 was involved in the Akt-mTOR and PI3-MAPK pathway to up-regulating myogenic genes of myoblasts under normoxic^7, 26^. In the hypoxic microenvironments, IGF2 can activate the MAPK/Erk pathway to promote cell proliferation and suppresses differentiation^7^. IGF2 also upregulated the phosphorylation of p-ERK1/2 and p-P38 MAPK in trophectoderm cells and promote the cell migration^27^. Blocking the IGF-II/IGF1R pathway reduced survival and clonogenicity of human ES cells. IGF-II was able to maintain the human ES cell cultures^11^. Interestingly, a report indicated that a constitutively activated IGF-IR support the transformation of immortalized human mammary epithelial cells and growth in immunocompromised mice^28^. In our study, we examined the p-Erk1/2 in medaka cells and p-Akt in 293 cells by western blot analysis, it indicated the medaka IGF2 and human IGF2 have the similar function to activate the phosphorylation of Erk1/2 and Akt.

Importantly, we examined the binding ability of IGF2:GFP on medaka blastomeres under fluorescent microscopy. Blastomeres isolated from the medaka embryos at midblastula stage were incubated with IGF2:GFP. By measuring the fluorescent intensity detected by fluorescent microscopy, we confirmed that IGF2:GFP can bind to medaka blastomeres. And the IC_50_ of this ligand was subsequently calculated by the competitive binding assay. As previously reports indicated that IGF2 could bind to several growth factor receptors^10^, the ligand binding ratio detected in our work could be considered as a generally quantified result. Additionally, the location of IGF2 on cell was examined under TEM, and it indicated that the IGF2 bond on the surface of blastomeres. Most importantly, we presented that the IGF2 sustained the viability and derivation of blastomeres in short-term cultured, which suggested that the IGF2 play an important role in primary cell culture.

In conclusion, we cloned medaka IGF2 cDNA and found the high homologies of medaka IGF2 with other species, revealing the conservation during its evolution. After optimizing the protein expression condition, we expressed medaka IGF2 and GFP-tagged IGF2 (IGF2:GFP) successfully. With this fused fluorescent protein tag, we developed a reliable method to verify the binding activity of IGF2 ligand to the cultured stem cells and blastomeres from fish embryos. Most importantly, we examined the bioactivity of IGF2 *in vitro* and it revealed that the IGF2 can sustain the self-renewal of embryonic stem cells of medaka and demonstrated the extensive application prospects of this ligand.

## Methods

### Fish and cells

Work with fish followed the guidelines on the Care and Use of Animals for Scientific Purposes of the National Advisory Committee for Laboratory Animal Research in Singapore and approved by this committee (permit number 27/09). Medaka fish was maintained under an artificial photoperiod of 14-h/10-h light/darkness at 26 °C and embryos were maintained at 28 °C as previously described^29^.

Medaka ES cell MES1^30^, haploid ES cell HX1^21^, spermatogonial cell SG3^31^ and zebrafish ES cell Z428^32^ were maintained at 28 °C in ESM4 medium as described^33, 34^, which contains high-glucose Dulbecco’s modified Eagle’s medium (DMEM; Invitrogen) supplemented with 20 mM HEPES (Sigma-Aldrich), 15% FBS (Invitrogen), 0.25 embryo/ml of medaka embryo extract (self-prepared), 2% of seabass serum (self-prepared), 10 ng/ml of human basic fibroblast growth factor (bFGF; PeproTech), 2 mM of L-glutamine (Invitrogen), 1 mM of nonessential amino acids (Invitrogen), 1 mM of sodium pyruvate (Invitrogen), (Invitrogen), 100 μM of 2-mercaptoethanol (Sigma-Aldrich), 2 nM of sodium selenite (Sigma) and 100 units/ml of penicillin-streptomycin. The 293 cells (85120602, Sigma) were cultured in high-glucose DMEM containing sodium pyruvate (110 mg/L), L-glutamine (200 mM) and 10% FBS in a 37 °C incubator with 5% CO_2_.

### Plasmid

First strand cDNA libraries were synthesized from total RNA isolated from the embryos of medaka strain i^3^ with RACE cDNA Amplification Kit (BD BioSciences). The gene encoding proIGF-II was amplified by PCR with primers of IGF2F (GCGTCTGCCATGGAGATCCC) and IGF2R (CAGTTGGTGTTTACTCGCCG). The cloned gene was verified by DNA sequencing. The gene *igf2* encoding IGF2 mature peptide was PCR-amplified by using a primer pair of IGF2Nco (GCCATGGGGCTGGCCTCGGCGGAG and IGF2Xho (GCTCGAGTTCCGACTTGGTGGGTT). This PCR product was digested and ligated in-frame within NcoI and XhoI sites of pET32a to generated plasmid pIGF2. To construct plasmid pIGF2gfp expressing EGFP fused IGF2 (IGF2:GFP), the *igf2* gene was PCR-amplified with a primer pair of IGF2Nco plus IGF2Hind (GAAGCTTTTCCGACTTGGTGGG) and ligated into pET32a together with an *egfp* gene amplified with primer pair of GFPhind (GAAGCTTGTGAGCAAGGGCGAGGAG) plus GFPXho (CCTCGAGCTTGTACAGCTCGTCC). Plasmid pGFP was constructed by insertion of a *gfp* gene within restriction sites of NcoI and XhoI in pET32a, which was PCR-amplified by GFPNco (ACCATGGTGAGCAAGGGCGAGG) plus GFPXho.

### Protein expression and purification

Plasmid pIGF2 was transformed into *E. coli* BL21 (DE3), and a single colony was cultured in 5 ml LB medium with ampicillin (100 µg/ml) overnight (37 °C, 200 rpm). Then the culture was inoculated into pre-warmed LB medium at a ratio of 1:50 and grew until OD_600_ of 0.5~0.7. Protein expression was induced overnight at 18 °C with IPTG ranged from 0.2 mM to 1 mM. After induction, cells were collected by centrifugation at 4,000 × g for 10 min and resuspended in 10 ml lysis buffer (50 mM NaH_2_PO_4_, 300 mM NaCl, 10 mM imidazole). The suspended cells were lysed by sonication on ice. Target protein in the supernatant was mixed with Ni-NTA agarose (Novagen) and purified under native condition. Purified fusion protein Trx:IGF2 was concentrated with ultrafiltration (10 kDa, Amicon) and resuspended in S-protein Agarose binding/wash buffer (150 mM NaCl, 20 mM Tris-HCl, 0.1% Triton X-100) to give a final concentration of 2 mg/ml. Then the S-protein Agarose (Novagen) was added at the ratio of 500 µg Trx:IGF2 per milliliter of agarose and incubated for 1 h at room temperature. After washing with binding/wash buffer, enterokinase (NEB) was added to the mixture at the ratio of 0.6 ng enzymes per 100 µg fusion protein and incubated for 8 h at 25 °C. The target protein of recombinant medaka IGF2 was finally eluted from the agarose. The IGF2:GFP and GFP protein was expressed and purified individually following the same procedure.

### Cell surface binding assay

The binding of IGF2:GFP to cultured stem cells was examined by flow cytometry analysis. To minimize the effects of growth factors contained in ESM4 medium, cells were washed with phosphate-buffered saline (PBS) and starved for 8 h in the basic medium without bFGF, FBS, embryo extract and seabass serum but with supplemented 5% BSA. The cells were trypsinized and suspended at the concentration of 10^5^ cells/ml and the IGF2:GFP was added into cell suspension at the concentration of 100 nM and subsequently incubated for 30 min at 4 °C. After washing with PBS thoroughly, cells were counterstained with Hoechst 33342 at 2 μg/ml. The cells incubated with IGF2:GFP and the cells incubated with or without GFP (100 nM) as control were analyzed on the BD LSR Fortessa cell analyzer respectively (Becton Dickinson, San Jose, CA, USA).

Medaka blastomeres from the midblastula stage embryos were dissected in pure DMEM. About 1000 living or fixed (2% PFA fixed for 5 min and washed thoroughly) blastomeres suspended in 0.5 ml PBS were incubated with IGF2:GFP at the final concentration of 100 nM for 10 min at 4 °C and washed 3 times with PBS. Meanwhile, cells incubated with GFP at the same concentration were set as a parallel control. The competitive binding assay was performed by co-incubated the cells with the IGF2:GFP at 500 nM and IGF2 at the increasing concentration from 1 nM to 1 µM. After washing, cells were checked under fluorescent microscopy immediately (Axioskop2plus, Zeiss) and about 100 cells in total were photographed on random sections from each treatment. Mean Fluorescence intensity (MFI) per cell was calculated with Image-Pro software and expressed in arbitrary units (AU).

### Cell viability assay

The HX1 cells cultured in a 24-well plate (10^5^/well) were washed with PBS and starved in basic medium for 8 h. After starvation, cells were cultured in basic medium for 2 days with supplemented IGF2, IGF2:GFP and human recombinant IGF2 (h-IGF2, I2526, Sigma) ranging from 0 to 200 nM respectively. The cells in the control group were continuously cultured in ESM4 medium. The morphologies of cultured cells were recorded with a microscope and the cell viability was assayed with AlamarBlue (Thermo, USA) according to the manufactory instruction. In detail, 100 µl of reagent was added to each well and incubated for 2 h at 28 °C. Then, 200 µl of supernatant from each well was transferred into a 96-well black bottom plate (Greiner, USA) and measured by a fluorometer (Cytation 3, USA) with excitation at 550 nm and emission at 590 nm. Results were presented in relative fluorescence intensity (FI).

### Western blot analysis

HX1 cells cultured in 6-well plate were starved for 8 h and subsequently incubated with growth factors of IGF2, IGF2:GFP and h-IGF2 respectively at the concentration of 200 nM for 1 h. The cells were washed with pure DMEM and lysed with 250 µl RIPA lysis buffer (Cat. 89900, Thermo) with additional protease Inhibitor (Cat. 78430, Thermo). After heating for 10 min at 95 °C with 50 µl SDS loading buffer, 15 µl of protein lysate was loaded for SDS-PAGE electrophoresis and the following western blot analysis. Membranes with target proteins were blocked for 60 min with 5% bovine serum albumin (BSA) in TBST (10 mM Tris-HCl (pH 7.9), 150 mM NaCl, 0.1% Tween-20). The Erk2 antibody (Cat. SC-1647, Santa Cruz), p-Erk1/2 antibody (Cat. 9101, Cell Signaling), Akt antibody (Cat. 9272, Cell Signaling), p-Akt antibody (Cat. 9271, Cell Signaling) and antibody against β-Actin (clone AC-74, Sigma) was incubated with the membrane respectively as primary antibody following previously reported^35^.

### RT-PCR analysis

After culturing for 2 days with IGF2 added at different concentrations, HX1 cells were collected and the RNA was extracted from each treatment for RT-PCR analysis performed as previously described^31, 36^. PCR was run in a 20-µl volume containing 10 ng of cDNA reaction for 25 (β-actin as an internal control) and 30 or 35 cycles (95 °C for 30 sec, 60 °C for 20 sec and 72 °C for 1 min; other genes). PCR products were separated on 1.5% agarose gels. Primers are listed in Supplementary Table S1.

### Location of the IGF2 under TEM

To precisely locate the growth factor on blastomeres, the cells were incubated with nanogold conjugated IGF2 and sampled for TEM observation. In detail, the histidine-tagged IGF2 was incubated with 1.8 nm Ni-NTA-Nanogold according to the instruction (Cat. 2080, Nanoprobe), and the conjugated protein was examined with SDS-PAGE electrophoresis. To visualize the protein bands, the gel was stained by Pageblue (Cat. R0571, Fermentas) and nanogold enhancement reagents following the instruction (Cat. 2115, Nanoprobe). Subsequently, the IGF2-nanogold conjugants were purified with gel filtration by using GE AKTA FPLC system. After purification, the IGF2-nanogold was incubated with blastomeres for 10 min at 4 °C with the concentration of 100 nM. The blastomeres incubated with nanogold at the same concentration were taken as control. Then the blastomeres were fixed by high pressure freezing for TEM sample preparation according to the previously report^37^. In order to enlarge the gold particles for TEM detection, the ultrathin sections (120 nm thick) were picked up on 50-mesh nickel grids for gold enlargement according to the instruction (Cat. 2113, Nanoprobe).

### Short-term primary culture

In order to test the bioactivity of IGF2, the medaka embryos at the midblastula stage were dissected and the blastomeres were seeded in basic medium containing IGF2 for short-term primary culture as a previously report^38^. In detail, fertilized embryos were incubated in PBS containing proteinase K (2 mg/ml) at 28 °C until the midblastula stage. The embryos were rinsed twice in PBS and sterilized in PBS-0.1% bleach for 2 min, and rinsed 5 times in PBS. The blastomeres were carefully dissected out from embryos and dissociated by gently pipetting. The blastomeres were rinsed 5 times by partial PBS changes and seeded into gelatin-coated 24-well plates containing 300 μl ESM4 or defined medium containing IGF2 at the concentration ranged from 0 to 200 nM. Approximately 2500 blastomeres were seeded into each well, and the cells were monitored under invert microscopy with phase contrast for counting the living and dead cells after 5 h incubation. To further identify the necrotic/dead cells, cells were stained with propidium iodide (PI, 1 ug/ml) and checked under fluorescent microscopy. After incubation for 18 h, the unattached cells were suspended and transferred into a 24-well plate for counting. Meanwhile, the attached cells in the original well were also counted to calculate the ratio of attachment.

### Statistical analysis

The Dunnett’s test was conducted by using GraphPad Prism v4.0. Data are presented as means ± S.D, and P < 0.05 were calculated by using Student’s t-test and considered as significant differences as described^34^.

## Electronic supplementary material

Supplementary Information
