# Supplementary material for: Medaka insulin-like growth factor-2 supports self-renewal of the embryonic stem cell line and blastomeres in vitro
Source: Sci Rep. 2017 Mar 6;7:78. doi: 10.1038/s41598-017-00094-y (PMC5428361; doi:10.1038/s41598-017-00094-y)
Supplement: Supplementary file 1 — Supplementary Information [file 41598_2017_94_MOESM1_ESM.pdf]

Medaka insulin-like growth factor-2 supports self-renewal of the embryonic stem cell line and blastomeres *in vitro*

Yongming Yuan, Yunhan Hong*

Department of Biological Sciences, National University of Singapore, Singapore 117543, Singapore

*Correspondent author: Professor Yunhan Hong

Department of Biological Sciences

National University of Singapore

Singapore 117543

E-mail: dbshyh@nus.edu.sg

Tel: +65-65162915; Fax: +65-67792486

Supplementary files list:

I. Supplementary table

Table S1. Genes and primers used for RT-PCR analyses

II. Supplementary figure legend

Figure S1. Morphology of HX1 cells cultured with the supplementary of IGF2:GFP or h-IGF2. HX1 cells were cultured for 2 days in basic medium with IGF2:GFP and h-IGF2 added respectively at varying concentrations. The morphology of cultured cells were examined. (**a**-**c**) Micrographs of HX1 cells cultured in medium containing IGF2:GFP at indicated concentration. (**d**-**f**) Micrographs of HX1 cells cultured in medium containing h-IGF2 at indicated concentration. Scale bars, 5 μm.

Figure S2. Specific detection of the phosphorylated MAPK/Erk and Akt in cells. 293 cells and HX1 cells cultured in complete medium (lane 1 and 3) and basic medium DMEM (lane 2 and 4) respectively were sampled for western blot analysis to detected the phosphorylated Erk and Akt in cells. The phosphorylation of Erk1/2 (p-Erk1/2) were examined with phospho-Erk1/2 antibody and the phosphorylation of Akt (p-Akt) were detected by phospho-Akt antibody. Antibody against Erk2 and Akt was used to identify the intracellular Erk2 and Akt respectively.

Supplementary table

Table S1. Genes and primers used for RT-PCR analyses

Gene	Accession number	Primer	Sequences (5' to 3')	Size (bp)
oct4	AY639946	oct4F	GTAGGTCACCTGACAGGATG	660
		oct4R	CTGATTGCACTCTGACAGC	
nanog	FJ436046	nanogF	ATGGTTGAGTCCCAATC	321
		nanogR	ATATCGCTCTGAAACCCAG	
brachyury (ntl, no tail)	ENSORLG000000 11262	ntlF	CTGCCTACCAGAACGAAGAGA	985
		ntlR	TTCGATCAGTAGAAGGCACGT	
IGF-1R	ENSORLT000000 18467.1	IGF-1RF	CGACTCCTGGGTGTGGTCTC	379
		IGF-1RR	GAGACTCGGGCGACATCCAGC	
β-actin	S74868	ActinF	TTCAACAGCCCTGCCATGTA	650
		ActinR	CCTCCAATCCAGACAGTAT	

Supplementary figures

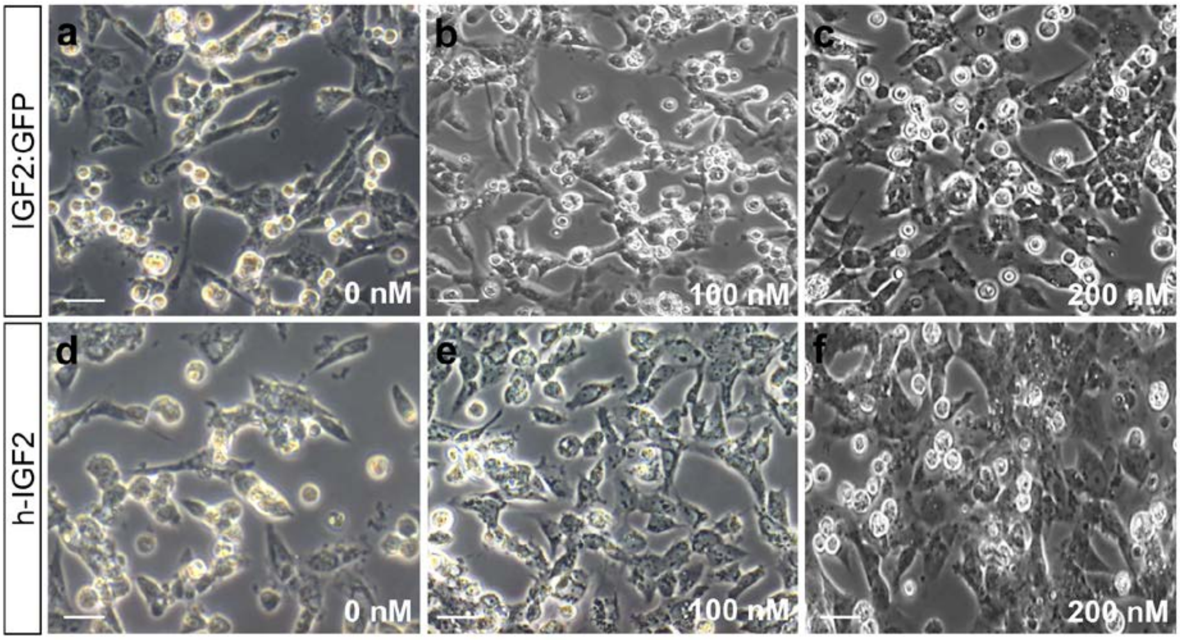

Figure S1. Morphology of HX1 cells cultured with the supplementary of IGF2:GFP or h-IGF2. HX1 cells were cultured for 2 days in basic medium with IGF2:GFP and h-IGF2 added respectively at varying concentrations. The morphology of cultured cells were examined. (**a-c**) Micrographs of HX1 cells cultured in medium containing IGF2:GFP at indicated concentration. (**d-f**) Micrographs of HX1 cells cultured in medium containing h-IGF2 at indicated concentration. Scale bars, 5 μm.

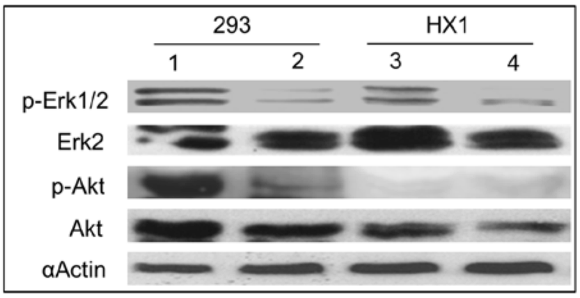

Figure S2. Specific detection of the phosphorylated MAPK/Erk and Akt in cells. 293 cells and HX1 cells cultured in complete medium (lane 1 and 3) and basic medium DMEM (lane 2 and 4) respectively were sampled for western blot analysis to detected the phosphorylated Erk and Akt in cells. The phosphorylation of Erk1/2 (p-Erk1/2) were examined with phospho-Erk1/2 antibody and the phosphorylation of Akt (p-Akt) were detected by phospho-Akt antibody. Antibody against Erk2 and Akt was used to identify the intracellular Erk2 and Akt respectively.
